# Between semelparity and iteroparity: Empirical evidence for a continuum of modes of parity

**DOI:** 10.1002/ece3.3341

**Published:** 2017-09-07

**Authors:** Patrick William Hughes

**Affiliations:** ^1^ Department of Plant Breeding and Genetics Max Planck Institute for Plant Breeding Research Köln Germany

**Keywords:** annual, iteroparity, life history, parity, perennial, phenology, reproduction, semelparity

## Abstract

The number of times an organism reproduces (i.e., its mode of parity) is a fundamental life‐history character, and evolutionary and ecological models that compare the relative fitnesses of different modes of parity are common in life‐history theory and theoretical biology. Despite the success of mathematical models designed to compare intrinsic rates of increase (i.e., density‐independent growth rates) between annual‐semelparous and perennial‐iteroparous reproductive schedules, there is widespread evidence that variation in reproductive allocation among semelparous and iteroparous organisms alike is continuous. This study reviews the ecological and molecular evidence for the continuity and plasticity of modes of parity—that is, the idea that annual‐semelparous and perennial‐iteroparous life histories are better understood as endpoints along a continuum of possible strategies. I conclude that parity should be understood as a continuum of different modes of parity, which differ by the degree to which they disperse or concentrate reproductive effort in time. I further argue that there are three main implications of this conclusion: (1) that seasonality should not be conflated with parity; (2) that mathematical models purporting to explain the general evolution of semelparous life histories from iteroparous ones (or vice versa) should not assume that organisms can only display either an annual‐semelparous life history or a perennial‐iteroparous one; and (3) that evolutionary ecologists should base explanations of how different life‐history strategies evolve on the physiological or molecular basis of traits underlying different modes of parity.

## 1 INTRODUCTION

Semelparity (and the related botanical term “monocarpy”) describes the life history defined by a single, highly fecund bout of reproduction, and can be contrasted with iteroparity (“polycarpy”), the life history defined by repeated (i.e., “iterative”) bouts of reproduction throughout life. Identifying the reasons why organisms adopt either mode of parity is one of life‐history theory's oldest problems, having been considered by both Aristotle (History of Animals, BkIX, 622 1‐30, trans. Thompson, 1907) and Linnaeus (Linnaeus, 1744). In contemporary evolutionary ecology, this problem has been formalized by age‐structured demographic models that seek to explain the eco‐evolutionary dynamics of reproductive patterns by comparing the intrinsic rates of increase (i.e., density‐independent growth rates) of reproductive strategies (Bryant, 1971; Charnov & Schaffer, 1973; Cole, 1954; Cushing, 2015; Javoiš, 2013; Omielan, 1991; Su & Peterman, 2012; Vaupel, Missov & Metcalf, 2013; Young, 1981). In such models, two modes of parity are considered, classified by whether they express all reproductive effort in a single year (semelparity), or in more than one (iteroparity). Here, I refer to this simplified conception as the “discrete conception of parity.” The main advantage of the discrete conception of parity is its analytical simplicity; given population growth data, intrinsic rates of increase can be easily computed and directly compared. Some intraspecific comparisons between phenotypically similar semelparous and iteroparous congeners conform to the predictions of demographic models based on the discrete conception of parity (Fritz, Stamp & Halverson, 1982; Iguchi & Tsukamoto, 2001; Young, 1984, 1990).

However, in this review I will argue that despite the successes—both theoretical and empirical—of evolutionary explanations rooted in the discrete conception of parity, there is widespread evidence that, like many other life‐history traits, parity is a continuous variable and that semelparity and iteroparity are the endpoints of a continuum of possible strategies that define the distribution of reproductive effort through time, rather than simple alternatives describing whether an organism fatally reproduces in a given year or not. On this account, semelparity can be understood as the strategy defined by concentrating reproductive effort in time and iteroparity as the strategy defined by distributing reproductive effort over longer timescales. I refer to this idea hereafter as the “continuous conception of parity.” It is important to note that the continuous conception of parity should not be conflated with the related terms “annuality” and “perenniality.” These terms specify strategies defined by the “digitization” of reproduction in response to seasonal effects supervening on the process of reproduction, rather than describing how concentrated reproductive effort is in time. This distinction is further discussed later.

The abstract idea that parity itself is continuous and not discrete may be unpopular but is not new (Hughes & Simons, 2014c; Kirkendall & Stenseth, 1985; Roff, 1992; Unwin, Kinnison & Quinn, 1999). However, to date the degree to which empirical evidence supports the continuity of parity has not yet been examined. Furthermore, evolutionary explanations comparing life‐history differences between clades with differing modes of parity continue to rely on the discrete conception of parity (e.g., Lopes & Leiner, 2015), and mathematical models based on the formalization of this assumption continue to be produced (Benton & Grant, 1999; Davydova, Diekmann, and van Gils, 2005; Vaupel et al., 2013). However, because of the ubiquity of evolutionary transitions from iteroparity to semelparity (Table 1), understanding parity as a continuous trait is important for understanding the underlying eco‐evolutionary dynamics that affect the fitness of life‐history strategies.

**Table 1:** Angiosperm orders show substantial diversity in mode of parity. Data are shown for all 59 plant orders from the APG III system. Class = (M) monocot or (D) dicot. Marks indicate whether the indicated reproductive strategy is present in that order

Plant order	Families	Estimated number of species	Class	Exemplar species	Semelparous species present	Iteroparous species present	Notes	References
Acorales	1	2	M	*Acorus calamus*		x	–	Kew World Checklist (2017)
Alismatales	13	4,500	M	*Anthurium andraeanum*	x	x	Facultative semelparity	Haggard and Tiffney (1997), Haston et al. (2009)
Amborellales	1	1	–	*Amborella trichopoda*	x		–	Kew World Checklist (2017)
Apiales	7	5,500	D	*Daucus carota*	x	x	Facultative semelparity in Heracleum spp.	Chandler and Plunkett (2004), Kew World Checklist (2017)
Aquifoliales	5	600	D	*Ilex aquifolium*	x	x	–	Kew World Checklist (various authors) (2017)
Arecales	1	2,600	M	*Cocos nucifera*	x	x	Facultative semelparity in *Rhopalostylis sapida*	Silvertown et al. (1993)
Asparagarles	14	36,000	M	*Asparagus officinalis*	x	x	Facultative semelparity in *Yucca whipplei*	Keeley et al. (1986)
Asterales	11	27,500	D	*Helianthus annuus*	x	x	Facultative iteroparity in *Lobelia inflata*	Hughes and Simons (2014b,c)
Austrobaileyales	3	100	NA	*Illicium verum*		x	–	Palmer et al. (2004)
Berberidopsidales	2	6	D	*Berberidopsis corallina*		x	–	Kew World Checklist (various authors) (2017)
Brassicales	17	4,500	D	*Brassica oleracea*	x	x	“perpetual flowering” under genetic control (*Arabis alpina*); phenotypically plastic semelparity in *Brassica campestris*	Biswas and Mandal (1987), Wang, Farrona, Vincent, Fornara, et al. (2009), Albani et al. (2012), Kew World Checklist (various authors) (2017)
Bruniales	2	80	D	*Desfontainia spinosa*	x	x	Fire‐dependent facultative parity (Bruniaceae spp.)	van Wilgen and Forsyth (1992)
Buxales	3	120	D	*Buxus sempervirens*		x	–	Kew World Checklist (various authors) (2017)
Canellales	2	136	D	*Canella winteriana*	x	x	–	Kew World Checklist (various authors) (2017)
Caryophyllales	33	11,000	D	*Silene dioica*	x	x	Plasticity of iteroparous reproduction in *Ferocactus wislizeni*; variation in many long‐lived Cactaceae perennial spp.; similar changes in Buckwheat (Polygonaceae)	Bowers (2000), Song et al. (2013)
Celastrales	2	1,300	D	*Celastrus orbiculatus*		x	–	Kew World Checklist (various authors) (2017)
Ceratophyllales	1	10	–	*Ceratophyllum submersum*	x	x	Plasticity of iteroparous life histories in European hornworts	Bisang et al. (2008)
Chloranthales	1	75	–	*Sarcandra glabra*		x	–	Kew World Checklist (various authors) (2017)
Commelinales	5	850	M	*Commelina communis*	x	x	Phenotypically plastic semelparity in genus Commelina; varies from near‐uniparity to extended semelparity	Faden (1993, 2006)
Cornales	6	600	D	*Hydrangea macrophylla*	x	x	Facultative iteroparity in stickleaf spp.	Keeler (1987)
Crossosomatales	7	80	D	*Crossosoma bigelovii*		x		Kew World Checklist (various authors) (2017)
Cucurbitales	8	2,600	D	*Begonia obliqua*	x	x	Facultative iteroparity among cultivated begonias	De Wilde (2010)
Dilleniales	1	400	D	*Hibbertia stellaris*	x	x	Facultative semelparity in Hibbertia spp	Stebbins and Hoogland (1976)
Dioscoreales	3	1,050	M	*Dioscorea rotundata*		x	–	Kew World Checklist (various authors) (2017)
Dipsacales	2	1,100	D	*Lonicera periclymenum*		x	–	Kew World Checklist (various authors) (2017)
Ericales	25	11,000	D	*Vaccinium macrpcarpon*	x	x	Facultative semelparity and facultative iteroparity in Impatiens spp.	Vervoort et al. (2011)
Escalloniales	1	130	D	*Escallonia bifida*	x	x	–	Kew World Checklist (various authors) (2017)
Fabales	4	20,000	D	*Pisum sativum*	x	x	Facultative iteroparity in many spp.	Nichols et al. (2007)
Fagales	7	2,000	D	*Quercus alba*		x	–	Kew World Checklist (various authors) (2017)
Garryales	2	18	D	*Garrya congdonii*		x	–	Kew World Checklist (various authors) (2017)
Gentianales	5	16,000	D	*Gentiana verna*	x	x	Plasticity of iteroparous reproduction in long‐lived perennial *Frasera caroliniensis*	Threadgill et al. (1981)
Geraniales	5	900	D	*Geranium rotundifolium*	x	x	–	Kew World Checklist (various authors) (2017)
Gunnerales	2	55	D	*Gunnera manicata*	x	x	Facultative semelparity in *Gunnera herteri*	Wanntorp et al. (2002)
Huerteales	4	20	D	*Huertea cubensis*		x	–	Kew World Checklist (various authors) (2017)
Lamiales	20	24,000	D	*Lamium purpureum*	x	x	–	Kew World Checklist (various authors) (2017)
Laurales	7	2,500	D	*Laurus nobilis*		x	–	Kew World Checklist (various authors) (2017)
Liliales	10	1,300	M	*Lilium candidum*	x	x	Many perennial Agave spp. Have substantial phenotypic plasticity in parity	Nobel (1977), Arizaga and Ezcurra (1995), Rocha et al. (2005)
Magnoliales	6	5,000	–	*Magnolia virginiana*	x	x	–	Kew World Checklist (various authors) (2017)
Malpighiales	35	16,000	D	*Malpighia glabra*	x	x	–	Kew World Checklist (various authors) (2017)
Malvales	9	6,000	D	*Malva sylvestris*	x	x	–	Kew World Checklist (various authors) (2017)
Myrtales	9	11,000	D	*Myrtus communis*	x	x	–	Kew World Checklist (various authors) (2017)
Nymphaeales	3	70	–	*Nymphaea lotus*		x	–	Kew World Checklist (various authors) (2017)
Oxalidales	7	1,800	D	*Oxalis acetosella*	x	x	–	Kew World Checklist (various authors) (2017)
Pandanales	5	1,300	M	*Pandanus utilus*		x	–	Kew World Checklist (various authors) (2017)
Paracryphiales	1	36	D	*Paracryphia alticola*		x	–	Kew World Checklist (various authors) (2017)
Petrosaviales	1	5	M	*Petrosavia sakuraii*		x	–	Kew World Checklist (various authors) (2017)
Picramniales	1	65	D	*Picramnia xalapensis*		x	–	Kew World Checklist (various authors) (2017)
Piperales	4	4,000	–	*Piper nigrum*	x	x	Facultative semelparity in *Piper pellucida*	Wanke et al. (2006)
Poales	16	18,000	M	*Zea mays*	x	x	Facultative iteroparity and variable iteroparity in bamboo, wheat	Franklin (2004), Montti et al. (2011), Baum et al. (2013)
Proteales	3	1,000	D	*Protea caffra*		x	–	Kew World Checklist (various authors) (2017)
Ranunculales	7	2,800	D	*Ranunculus occidentalis*	x	x	Phenotypically plastic parity in many Meconopsis spp.	Sulaiman and Babu (1996)
Rosales	9	7,700	D	*Rosa blanda*	x	x	–	Kew World Checklist (various authors) (2017)
Sabiales	1	100	D	*Sabia campanulata*		x	–	Kew World Checklist (various authors) (2017)
Santalales	7	1,000	D	*Santalum ellipticum*		x	–	Kew World Checklist (various authors) (2017)
Sapindales	9	5,700	D	*Acer saccharum*		x	–	Kew World Checklist (various authors) (2017)
Saxifragales	16	2,500	D	*Saxifraga stellaris*	x	x	*Saxifraga longifolia* shows phenotypically plastic parity	García (2003)
Solanales	5	4,000	D	*Solanum tuberosum*	x	x	Plasticity of long‐term monocarpic reproduction in Petunia spp.	Laroche and Bousquet (1999)
Trochodendrales	1	2	D	*Trochodendron aralioides*		x	–	Kew World Checklist (various authors) (2017)
Vitales	1	770	D	*Vitus vinifera*		x	–	Kew World Checklist (various authors) (2017)
Zingiberales	8	2,100	D	*Zingiber officianale*	x	x	Plasticity of mode of parity in Ensete spp.	Kirchoff (1992), Birmeta et al. (2004)
Zygophyllales	2	300	D	*Zygophyllum album*	x	x	–	Kew World Checklist (various authors) (2017)

In this review, I begin by reviewing the development of both the discrete and continuous conceptions of parity as evolutionary hypotheses and/or models. Next, I review empirical work that highlights the existence of natural variation in reproduction along a semelparity–iteroparity continuum, focusing on three distinct patterns found in natural populations that are neither abstractly semelparous nor iteroparous: facultative iteroparity, facultative semelparity, and multiple modes of parity. I conclude by exploring the implications of the continuous conception of parity for: (1) the study of seasonality as a “digitization” of reproduction, (2) the process of mathematically modeling life‐history optimization, and (3) the study of the molecular regulation of reproductive traits linked to parity.

## 2 THE DISCRETE CONCEPTION OF PARITY

### 2.1 “Cole's Paradox” and the development of the discrete conception of parity

Although the first mathematical model of the intrinsic rate of increase in annual plants was constructed by Linnaeus (1744), Lamont Cole (1954) was the first to categorize life histories into dichotomous “semelparous” and “iteroparous” groups: A semelparous organism is one that “dies upon producing seed” and therefore, “potential population growth may be considered on the assumption that generations do not overlap” (p. 109), while iteroparous organisms include a variety of cases, from those where “only two or three litters of young are produced in a lifetime” as well as “various trees and tapeworms, where a single individual may produce thousands of litters” (p. 118). Thus, Cole created, and contemporary theorists have inherited, a conception of parity as a discrete variable: An organism either reproduces more than once or it does not.

Cole also identified “the paradox of semelparity,” and wrote that “for an annual species, the absolute gain in intrinsic population growth which could be achieved by changing to the perennial reproductive habit would be exactly equivalent to adding one individual to the average litter size.” (Cole, 1954, p. 118). Consequently, according to the model he developed, a semelparous or iteroparous strategy evolves in response to strong directional selection for trait values that: (1) maximize the annual rate of intrinsic increase; and (2) are subject to trade‐offs, since reproductive effort is always limited by resource availability. The “paradox of semelparity” is that the relative intrinsic rates of increase for semelparous and iteroparous strategies are very similar (i.e., they differ only by one individual—the mother), which suggests that iteroparity, not semelparity, should be rare, while in nature, iteroparous life histories are generally more common than semelparous ones. Cole's articulation of the paradox of semelparity motivated many studies searching for theoretical selective advantages of traits linked to discrete semelparous and iteroparous strategies (Cushing, 2015; Murdoch, 1966; Murphy, 1968; Omielan, 1991; Su & Peterman, 2012; Vaupel, Missov, and Metcalf, 2013), as well as attempts to detect these selective advantages in natural systems (Fisher & Blomberg, 2011; Franklin & Hogarth, 2008; Gagnon & Platt, 2008; Kaitala, Tesar & Ranta, 2002; Kraaijeveld, Kraaijeveld‐Smit & Adcock, 2003; Murphy & Rodhouse, 1999). Following Cole, semelparous strategies considered in later life‐history models were usually also annual (García, 2003; Young & Augspurger, 1991), and thus, the primary goal of many models purporting to explain the evolution of semelparity was to provide reasons why a perennial‐iteroparous strategy might confer higher fitness than an annual‐semelparous one.

Cole's “paradox of semelparity” was resolved by acknowledging that differences in age‐specific rates of mortality affect the relative fitness of semelparous and iteroparous habits. Building on prior analytical work (Bryant, 1971; Emlen, 1970; Gadgil & Bossert, 1970; Murphy, 1968), Charnov and Schaffer (1973) and Schaffer (1974b) noted that the expected fitness value of individuals at juvenile (i.e., prereproductive) and adult (i.e., reproductively mature) developmental stages often differed. They then argued that when the survival of adults was more assured than the survival of juveniles, an iteroparous habit would have a comparative growth advantage over a semelparous one. Thus, their model emphasized that the reproductive value of members of the age class with a lower age‐ or stage‐specific rate of mortality would be—assuming equal fitness across age classes—greater than the value of the members of the age class with a higher rate of mortality. This approach can also be used to analyze the age structures of iteroparous populations; thus, it is a “discrete” rather than a “binary” model. Young (1981) extended this insight into a more general model of intrinsic rates of increase, which incorporated not only differences in age‐specific survivorship, but also differences in prereproductive development time and time between reproductive episodes. This model provided three major reasons why semelparity might be favored by natural selection. First, high adult mortality—or the early onset of reproductive senescence—might prevent iteroparous species from accruing fitness gains from established parents over long timescales. Second, a high population growth rate should favor semelparity outright. Third, when the marginal cost of additional offspring is inversely proportional to the number of offspring produced, fecundity is maximized by investing all reproductive effort into a single episode, that is, adopting an extreme annual‐semelparous life history—see also Schaffer (1974a, 1974b) and Schaffer and Gadgil (1975).

### 2.2 Further theoretical work on parity as a discrete trait

Given that earlier work sought to explain the prevalence of semelparous and iteroparous strategies by identifying differences in age‐specific mortality, recent work has sought to explain why differences in age‐specific mortality persist, as well as how varying environmental conditions facilitate the co‐existence of different modes of parity. Models used to predict age and size at first flowering for semelparous plants have been found to be more appropriate for long‐lived than short‐lived species (Metcalf, Rose & Rees, 2003; Rees and Rose 2002). More recently, mathematical modeling of evolutionary responses to discrete semelparous and iteroparous strategies has shown that the maintenance of both modes of parity can be a consequence of stochasticity in the ratio of juvenile to adult mortality (Murphy, 1968; Ranta, Tesar & Kaitala, 2002), of differences in the effects of density on age‐specific mortality (Bulmer, 1985, 1994), or as a consequence of population instability (Ranta, Kaitala, Alaja & Tesar, 2000). Another common approach has been to use simulations, based on comparisons between discrete strategies, to argue that spatial heterogeneity and stochastic events (i.e., demographic disasters and windfalls) influence the evolutionary stability of each mode of parity over small spatial scales (e.g., Ranta, Tesar & Kaitala, 2001). Similarly, Zeineddine and Jansen (2009) examined the role that discrete modes of parity may play in evolutionary tracking, suggesting that species adopting an annual‐semelparous strategy may have an evolvability advantage over perennial–iteroparous congeners. Moreover, considerable evidence now supports two general conclusions: (1) that optimizing growth, reproduction, and phenology depend on optimizing parity (Iguchi & Tsukamoto, 2001; Keeley & Bond, 1999; Kraaijeveld et al., 2003; Leiner, Setz & Silva, 2008; Maltby & Calow, 1986; Stegmann & Linsenmair, 2002; Trumbo, 2013) and also (2) that parity is especially important for predicting reproductive scheduling (Cooke, Hinch, Farrell, Lapointe & Jones, 2004; Iwasa, 1991; Kozłowski, 1992; Kozłowski & Wiegert, 1986; McNamara, 1997; Miller, Williams, Jongejans, Brys & Jacquemyn, 2012; Oizumi, 2014; Schaffer and Gadgil 1975; Vaupel, Missov, and Metcalf, 2013), programmed senescence (Panagakis, Hamel & Cote, 2017; Ricklefs, 2008; Weimerskirch, 1992), and/or the optimal allocation of reproductive effort to offspring (Cohen, 1966; Einum & Fleming, 2007; Gremer & Venable, 2014; Mironchenko & Kozłowski, 2014; Smith & Fretwell, 1974; Winkler & Fischer, 2002). Thus, contemporary work in evolutionary ecology is replete with papers discussing the knock‐on effects of the assumption that modes of parity are discrete rather than continuous.

### 2.3 Empirical support for the discrete conception of parity

Empirical support for the predictions made by discrete‐conception models is strongest where perennial‐iteroparous and annual‐semelparous (or, rarely, perennial‐semelparous) congeneric species coexist and have starkly different life histories. For instance, in a comparison of Mount Kenya species of the genus *Lobelia*, Young (1984) found that juvenile and adult mortality of the annual‐semelparous species *L. telekii* were higher than in the closely related perennial‐iteroparous species *Lobelia deckenii* (syn. *L. keniensis*). Young concluded that the difference in age‐specific rates of mortality would strongly influence the expected value of future reproduction for each species, leading to perennial‐iteroparity in one species and annual‐semelparity in the other (see also Young, 1990). Similar comparisons between semelparous and iteroparous congeners or confamilials have been conducted in insects (Fritz et al., 1982; Stegmann & Linsenmair, 2002), salmon (Crespi & Teo, 2002; Dickhoff, 1989; Kindsvater, Braun, Otto & Reynolds, 2016; Unwin, Kinnison, and Quinn, 1999), snakes (Bonnet, 2011), algae (De Wreede & Klinger, 1988), and dasyurid marsupials (Kraaijeveld, Kraaijeveld‐Smit, and Adcock, 2003; Mills, Bradshaw, Lambert, Bradshaw & Bencini, 2012). Other studies have focused on reproductive effort, as a declining marginal cost of offspring in terms of reproductive effort should select for an annual‐ or perennial‐semelparous life history over an perennial‐iteroparous one. This is the cited cause of the evolution of semelparity in *Digitalis purpurea* (Sletvold, 2002), and in *Antechinus agilis* (Fisher & Blomberg, 2011; Smith & Charnov, 2001). The interaction between intrinsic rate of increase and phenology also has important fitness implications; in two subspecies of *Yucca whipplei*, the semelparous variant showed higher viability and faster time to germination than the iteroparous variant did (Huxman & Loik, 1997). Further studies highlight the mortality differences between juveniles and adults, which explains the evolution of semelparity in a variety of long‐lived semelparous plants (Foster, 1977; Kitajima & Augspurger, 1989; Young & Augspurger, 1991), as well as in salmonids (Crespi & Teo, 2002; Fleming, 1998; Hendry, Morbey, Berg & Wenburg, 2004; Sloat et al., 2014). Taken together, even when not biologically plausible, conceptual models have proven to be heuristically valuable and have been used to draw stark contrasts between the different effects density dependence has on annual‐semelparous and perennial‐iteroparous strategies. In cases where such extreme strategies coexist, existing theories seem to do a good job of predicting how they evolved.

## 3 THE CONTINUOUS CONCEPTION OF PARITY

### 3.1 From uniparity to continuous reproduction

However, in many cases substantial unexplained variation in parity exists even after factors such as age‐specific mortality, density dependence, and environmental effects are taken into account. For this reason, it seems as though models based on the discrete conception of parity describe a limited range of special cases and not the majority of systems with congeneric or confamilial species with a spectrum of different reproductive strategies. This problem arises because theoretical models of the discrete conception of parity make two characteristic assumptions. First, it is assumed that reproductive output is allocated among cycles (typically seasons or years) rather than expressed continuously. This means that offspring produced at two different times within a single season are “counted” as being part of the same reproductive episode, while offspring produced at two different times in two different seasons are counted as part of categorically different reproductive episodes. This permits the calculation of threshold values (e.g., of size or age) beyond which selection should begin to favor one mode of parity or the other, but this is based on a distinction that is arbitrary. Second, each individual is assumed to express a single reproductive strategy; models do not predict phenotypically plastic modes of parity, or facultative switching between modes.

These assumptions do not hold in many cases. There are relatively few examples of semelparous reproduction occurring exactly “once”—that is, in exactly one place, at exactly one time. Moreover, “annuality” and “perenniality”—terms that refer to the number of years in which organisms reproduce—cannot be used interchangeably with “semelparity” and “iteroparity,” which refer to the number of reproductive episodes organisms have (Fritz, Stamp, and Halverson, 1982; Kirkendall & Stenseth, 1985). In “The Evolution of Life Histories”, Roff (1992) noted that, “if we consider our unit of time to be a single year, annuals can be termed semelparous and perennials iteroparous. A further division is possible within annuals, for some reproduce once and are, therefore, semelparous within any time scale, while others flower repeatedly throughout the summer and, hence, are iteroparous with respect to annuals that flower only once, but semelparous with respect to perennials” (p. 248). That is, it is the simultaneity and the finality of the reproductive episode (i.e., the concentration of reproductive effort) that defines “perfect” semelparity. Therefore, the continuous conception characterizes “extreme” semelparity to be a single, complete, and exhaustive reproductive episode where all reproductive effort is invested at once. Examples of this strategy—which Kirkendall and Stenseth (1985) termed “uniparity”— include mayflies and mites of the genus Adactylidium (Corkum, Ciborowski & Poulin, 1997; Edmunds, Jensen & Berner, 1976). Both male and female mayflies die shortly after mating and dispersing fertilized eggs. In Adactylid mites, offspring devour the mother from the inside out and are thus obligately annual‐semelparous (Elbadry & Tawfik, 1966; Goldrazena, Jordana & Zhang, 1997). The correspondingly “extreme” perennial‐iteroparous strategy is a long‐lived perennial strategy that spreads reproductive effort out evenly among a very large number of reproductive cycles. Many species, including bristlecone pine, many deep‐sea zoanthids, and other supercentennial species that reproduce regularly show such a habit (Baker, 1992; Druffel et al., 1995; Finch, 1998; Rozas, 2003). Intermediate strategies complete reproduction over a shorter timescale than bristlecone pine, but over a longer timescale than Adactylid mites.

The continuous conception of parity is therefore very simple: Parity should be understood as a composite trait, and, rather than considering only whether organisms complete reproduction within a given year, life‐history strategies should be compared by the degree to which they concentrate or disperse reproductive effort—and hence risk of reproductive failure—in time. For example, a mature biennial strategy (where an organism reproduces once per year in two consecutive years) distributes reproductive effort over a shorter timescale than does a long‐lived perennial congener (where an organism reproduces once per year in many years); although the biennial strategy is not semelparous, it is further toward the “uniparous” end of the continuum of modes of parity than is the perennial strategy. Similarly, an annual‐semelparous life history that reproduces rapidly lies further toward this end of the continuum than does an annual‐semelparous life history in which reproduction is spread over a longer period of time. In extending the underlying logic of the discrete conception in this way, the insights gained by comparing “extreme” semelparous and iteroparous strategies are included, but the explanatory power of this logic is extended to apply to intermediate strategies as well.

### 3.2 Empirical support for the continuous conception of parity

There is considerable empirical support, from laboratory and field studies alike, for the notion that parity varies continuously. Many species are facultatively semelparous, others reproduce irregularly or opportunistically, and many comparisons between related iteroparous and semelparous species do not show measurable differences in factors affecting intrinsic rates of increase, including age‐specific rates of mortality. These situations are not uncommon in nature. The problem they present is significant because the evolutionary transition from semelparity to iteroparity (and back) is ubiquitous, and has occurred in a wide variety of taxa (see Table 1 for an example using data from angiosperm orders).

There are important consequences for adopting the continuous conception of parity as a starting point for modeling the evolution of different modes of parity. Mathematical models based on the discrete conception of parity often predict threshold values—in mortality rate, size at initiation of reproduction, or expected growth rate—that do not agree with empirical observation (Lessells, 2005; Omielan, 1991; Piñol & Banzon, 2011; Su & Peterman, 2012; Trumbo, 2013; Vaupel, Missov, and Metcalf, 2013). In particular, ESS models derived from assumptions rooted in the discrete conception of parity frequently underestimate the adaptive value of semelparous reproductive strategies; even after accounting for the effects of environmental stochasticity and density dependence, ESS models predict that semelparous strategies should be less abundant—and less fit—than they have been found to be (Benton & Grant, 1999). In addition, there are empirical cases that explicitly do not conform to the predictions of the discrete model. For example, an analysis of 12 winter‐establishing primrose species (Oenothera: Onagraceae) found no significant differences in mortality estimates or in environmental determinants of fitness for semelparous and iteroparous species (Evans et al., 2005). In some cases, the problem may be that life histories are too complex for organisms to follow discrete strategies; many salmon species also do not fit neatly into “classical” annual‐ or perennial‐semelparous and perennial‐iteroparous classifications (Hendry et al., 2004; Unwin et al., 1999). Other research has suggested that deterministic models of investment may provide more accurate demographic predictions for long‐lived than short‐lived semelparous species, given that many annual‐semelparous species (usually plants) show substantial phenotypic plasticity in phenology (e.g., size at first flowering), offspring quality, and overall fecundity (Burd, Read, Sanson, Jaffre & Jaffré, 2006).

To provide a coherent exposition of the extensive body of recent work that shows empirical support for the continuous conception of parity, in what follows I focus on three “intermediate” life histories that are neither annual‐semelparous nor perennial‐iteroparous, but express another mode of parity that falls somewhere in between. These include the following: (1) facultative iteroparity; (2) facultative semelparity; and (3) multiple modes of parity expressed simultaneously. Although these three examples are the most common modes of parity that are neither classically (annual or perennial) semelparous nor iteroparous, other intermediate strategies exist, particularly when species or clades have idiosyncratic life histories. In addition, many differences in mode of parity are due to phenotypic plasticity; that is, life‐history strategies “intermediate” between annual‐semelparity and perennial‐iteroparity are displayed in response to an important environmental cue. For instance, polar cod (*Boreogadus saida*) are annual‐semelparous in nature, but can—given low extrinsic mortality—reproduce in two consecutive years in captivity, making them facultatively iteroparous (Hop and Gjøsæter, 2013; Hop, Trudeau & Graham, 1995). However, males and females of this species also seem to have different life histories—males begin to reproduce at an earlier age and can, in response to environmental stressors of varying strength, allocate varying (and even extreme) amounts of reproductive effort to a single instance of reproductive activity; parity in this species is thus also continuously varying and phenotypically plastic, and populations display multiple modes of parity at once (Nahrgang et al., 2014). Although examples of each life history are provided below, many more have been added to Table 2, a list of species showing facultatively varying, continuously varying and phenotypically plastic modes of parity. However, not all populations simultaneously expressing multiple modes of parity do so because of phenotypic plasticity; in many cases, this variation is the result of genetic differences between individuals (e.g., Hautekèete, Piquot, and Van Dijk, 2001; Leys et al., 2014). Finally, it may seem conceptually strange to present “facultative” life histories as evidence in favor of the continuous conception of parity, as the very logic of this conception speaks of a continuum of strategies, rather than a phenotypically plastic switch between discrete ones. I discuss facultative semelparity and iteroparity here for two reasons: first, these strategies are genuine examples of nondiscrete parity. They are often remarkable from a natural history viewpoint‐‐even if they do not show multiple modes of parity‐‐and are often reported in this way in the literature. Thus, this is an abundant source of empirical evidence that organisms do not show, as demographic models predict, only a single mode of parity. Second, mode of parity is often subject to a supervening effect of seasonality (see below for further discussion of this effect), and therefore, strategies intermediate between annual‐semelparity and biennial‐ or perennial‐iteroparity (for instance) may disappear not because there can only be two modes of parity, but rather because offspring are only viable when they are produced in certain seasons. Such seasonal effects are certainly important, but they do not arise strictly from differences in the intrinsic rate of increase. Species which show facultative switching may, where seasonal effects are less pronounced, show a wider range of possible modes of parity.

**Table 2:** Species known to display facultative semelparity, facultative iteroparity, a continuum of modes of parity, or phenotypic plasticity with respect to mode of parity

Focal species	Clade	Continuously varying traits identified	Facultative iteroparity	Facultative semelparity	Continuous variation in parity	Phenotypically plastic parity	References
*Acer negundo*	Angiosperm	Timing of reproduction				x	Lamarque et al. (2015)
*Agave celsii, Agave difformis*	Angiosperm	Timing of reproduction; reproductive effort			x	x	Rocha et al. (2005)
*Allogamus uncatus*	Insect	Timing of reproduction; duration of reproduction			x	x	Shama and Robinson (2009)
*Alosa sapidissima*	Fish	Timing of reproduction; clutch size		x	x	x	Leggett and Carscadden (1978)
*Amblyrhychus cristatus*	Reptile	Timing of reproduction			x	x	Vitousek et al. (2010)
*Antechinus stuartii*	Mammal	Adult mortality	x		x		Fisher and Blomberg (2011)
*Arabidopsis lyrata*	Angiosperm	Timing of reproduction; duration of reproduction			x		Remington et al. (2015)
*Arabis fecunda*	Angiosperm	Reproductive effort			x	x	Lesica and Shelly (1995)
*Bambusa narnhemica*	Angiosperm	Timing of reproduction			x		Franklin (2004)
*Beta vulgaris*	Angiosperm	Reproductive effort				x	Letschert (1993), Hautekèete et al. (2001, 2002, 2009)
*Boreogadus saida*	Fish	Adult mortality; reproductive effort			x		Nahrgang et al. (2014)
*Botryllus sclosseri*	Tunicate	Timing of reproduction, clutch size	x		x	x	Grosberg (1988), Harvell and Grosberg (1988)
*Bradybaena pellucida*	Gastropod	Duration of reproduction			x	x	Nyumura and Asami (2015)
*Cynodonichthys brunneus, Cynodonichthys magdalenae, Cynodonichthys kuelpmanni, Anablepsoides immaculatus,* and* Laimosemion frenatus*	Fish	Timing of reproduction; diapause length		x	x	x	Varela‐Lasheras and Van Dooren (2014)
*Daphnia galeata*	Crustacean	Timing of reproduction			x	x	Henning‐Lucass et al. (2016)
*Daucus carota*	Angiosperm	Timing of reproduction			x	x	Lacey (1988)
*Digitaria californica*	Angiosperm	Juvenile mortality			x		Smith et al. (2000)
*Dosidicus gigas*	Mollusk	Timing of reproduction; duration of reproduction	x		x	x	Hoving et al. (2013)
*Emoia atroscostata*	Reptile	Timing of reproduction			x		Alcala and Brown (1967)
*Erpobella octoculata*	Leech	Adult mortality; reproductive effort	x		x	x	Maltby and Calow (1986)
*Erysimum capitatum*	Angiosperm	Timing of reproduction		x	x		Kim and Donohue (2011)
*Eulamprus tympanum*	Reptile	Clutch size; timing of reproduction			x	x	Doughty and Shine (1997)
*Forficula auricularia*	Insect	Timing of reproduction				x	Meunier et al. (2012)
*Fucus serratus; Himanthalia elongata*	Algae	Duration of reproduction			x		Brenchley et al. (1996)
*Gaimardia bahamondei*	Bivalve	Timing of reproduction; duration of reproduction	x		x	x	Chaparro et al. (2011)
*Galaxias maculatus*	Fish	Timing of reproduction	x			x	Stevens et al. (2016)
*Gasterosteus aculeatus*	Stickleback	Timing of reproduction; reproductive effort		x	x	x	Snyder (1991), Bell and Foster (1994), Baker et al. (2008, 2015)
*Gracilinanus microtarsus*	Mammal	Male adult mortality	x		x	x	Kraaijeveld et al. (2003), Martins et al. (2006)
*Idiosepius pygmaeus*	Cephalopod	Timing of reproduction	x		x		Lewis and Choat (1993), Nesis (1996)
*Ligia cinerascens*	Crustacean	Adult mortality; timing of reproduction	x	x	x	x	Furota and Ito (1999)
*Lobelia inflata*	Angiosperm	Timing of reproduction; duration of reproduction	x		x	x	Hughes and Simons (2014a,b,c)
*Loligo vulgaris*	Cephalopod	Timing of reproduction; clutch size	x		x		Melo and Sauer (1999), Sauer et al. (1999)
*Mallotus villosus*	Fish	Adult mortality; timing of reproduction		x	x	x	Christiansen et al. (2008)
*Marmosops paulensis*	Mammal	Female adult mortality	x		x	x	Leiner et al. (2008)
*Mimulus guttatus*	Angiosperm	Duration of flowering			x	x	Van Kleunen (2007)
*Misumena vatia*	Arachnid	Juvenile mortality			x	x	Morse (1994), Morse and Stephens (1996)
*Nautilus* spp.	Cephalopod	Timing of reproduction; timing of senescence	x		x	x	Ward (1983, 1987)
*Oenothera deltoides; Oenothera pallida*	Angiosperm	Timing of reproduction; adult mortality	x	x	x		Evans et al. (2005)
*Oncorhymchus mykiss*	Fish	Reproductive effort	x		x	x	Seamons and Quinn (2010)
*Oncorhynchus nerka*	Fish	Timing of reproduction				x	Hendry et al. (2004)
*Oncorhynchus tshawytscha*	Fish	Adult mortality	x			x	Unwin et al. (1999)
*Onopordum illyricum*	Angiosperm	Timing of reproduction		x			Rees et al. (1999)
*Opisthoteuthis agassizii, Opisthoteuthis grimaldii* and *Grimpoteuthis glacialis*	Cephalopod	Timing of reproduction; reproductive effort			x		Aldred et al. (1983), Villanueva (1992), Vecchione et al. (1998)
*Panicum bisulcatum, Cyperus michelianus, Fimbristylis miliacea,* and *Eclipta prostrata*	Angiosperm	Timing of reproduction; duration of reproduction			x	x	Song et al. (2015)
*Parantechnius apicalis*	Mammal	Adult mortality		x		x	Wolfe et al. (2004)
*Plecoglossus altivelis*	Fish	Timing of reproduction; reproductive effort	x		x	x	Iguchi (1996), Iguchi and Tsukamoto (2001)
*Puya raimondii*	Angiosperm	Reproductive effort		x	x		Jabaily and Sytsma (2013)
*Rana arvalis*	Frog	Timing of reproduction				x	Richter‐Boix et al. (2014)
*Sasa senanensis, Sasa kurilensis,* and *Sasa palmata*	Angiosperm	Timing of reproduction; reproductive effort	x	x		x	Mizuki et al. (2014)
*Sepia officinalis*	Cephalopod	Adult mortality; timing of reproduction; clutch size; timing of senescence	x		x	x	Boletzky (1988), Rocha et al. (2001)
*Stegodyphus lineatus*	Arachnid	Adult mortality; timing of reproduction			x	x	Schneider and Lubin (1997)
*Sthenoteuthis oualaniensis*	Cephalopod	Timing of reproduction	x				Rocha et al. (2001)
*Strix uralensis*	Bird	Timing of reproduction				x	Brommer et al. (2012)
*Uta stansburiana*	Reptile	Duration of reproduction,		x	x	x	Tinkle (1969)
*Verbascum thapsis*	Angiosperm	Timing of reproduction			x		Reinartz (1984)
*Wyeomyla smithii*	Angiosperm	Timing of reproduction			x	x	Bradshaw (1986)
*Xerolenta obvia*	Gastropod	Timing of reproduction	x		x	x	Lazaridou and Chatziioannou (2005)
*Xeropicta derbentina*	Gastropod	Timing of reproduction	x		x	x	Aubry et al. (2005), Kiss et al. (2005)
*Yucca whipplei*	Angiosperm	Reproductive effort		x	x		Huxman and Loik (1997)

#### 3.2.1 Facultative iteroparity

Many semelparous species have shown the ability to facultatively reproduce one or more times after an initial bout of reproduction has begun and ended—this is termed “facultative iteroparity.” Facultative iteroparity can be adaptive when it either: (1) provides an opportunity to realize fitness gains from an unexpected abundance of resources, or (2) shifts reproductive effort from inopportune to opportune times. The first type of adaptive facultative iteroparity occurs when additional bouts of reproduction increase fitness by permitting unexpected “bonus” resources to be invested in new offspring. For example, mothers of the semelparous crab spider *Misumena vatia* (Araeae, Thomsidae) typically lay and provision a single brood of eggs (Gertsch, 1939; Morse, 1979); however, in response to high food availability and/or usually warm environmental conditions, they are capable of laying and caring for a second brood if sperm supplies are not depleted (Morse, 1994). A similar facultative double‐broodedness in response to unusually favorable environment has been observed in the green lynx spider *Peucetia viridans* (Fink, 1986). In addition, a small proportion of Chinook salmon (*Onchorhynchus tshawytscha*), which typically reproduce only once, have been found to survive and reproduce in two or three additional seasons (Unwin et al., 1999). Tallamy and Brown (1999) showed that large, well‐provisioned female burying beetles in multiple species in the genus Nicrophorus can reproduce more than once, despite the fact that small females can typically breed only once.

The second form of adaptive facultative iteroparity occurs when deferral of reproductive effort—from a primary reproductive episode to a secondary one—allows an organism to reproduce at a more opportune time. Reproduction is deferred to seek the highest marginal fitness return on invested reproductive effort. For example, when high organic pollution levels disrupt primary reproduction in the freshwater leech *Erpobdella octoculata*, reproduction ceases and remaining reproductive effort is deferred to a second reproductive bout produced the next year (Maltby & Calow, 1986). Similar behavior has been seen in another Erpobdellid leech, *Erpobdella obscura* (Davies & Dratnal, 1996; Peterson, 1983) as well as in many cephalopods (Rocha, Guerra & González, 2001). Adaptive deferral of reproductive effort is common in crab spiders. In *Lysiteles coronatus*, artificial brood reductions resulted in the production of a second brood, and the degree of deferral was proportional to the degree of the original reduction (Futami & Akimoto, 2005). This was also observed in the field in Eresid spiders of the genera Anelosimus and Stegodyphus, both of which facultatively produce a second brood in response to nest predation (Grinsted, Breuker & Bilde, 2014; Schneider & Lubin, 1997; Schneider, Salomon & Lubin, 2003). Although the adaptive potential of facultative iteroparity is often apparent, facultative iteroparity may also be vestigial instead of adaptive. In this case, the organism's life history merely reflects an ancestral state, and the second (or additional) bout of reproduction should confer little or no adaptive value (Golding & Yuwono, 1994; Hughes & Simons, 2014b).

#### 3.2.2 Facultative semelparity

Facultative semelparity occurs when species that are normally perennial‐iteroparous—that is, they have multiple, discontinuous reproductive episodes that span more than one year—are capable of expressing only a single reproductive bout (Christiansen, Præbel, Siikavuopio & Carscadden, 2008). This is a useful strategy for organisms to use to take advantage of unusually good environmental conditions for reproduction. For example, in the short‐lived mustard *Boechera fecunda* (syn. *Arabis fecunda*; Brassicaceae), plants are capable of wide range of reproductive strategies, from near‐instantaneous semelparity to multiyear iteroparity. This is because *B. fecunda* can produce many small axillary inflorescences in any given year, and their production does not preclude flowering by the same rosette in the subsequent year. However, plants can also produce large “terminal inflorescences” that exhaust remaining resources and lead to senescence and death. Although some plants produce axillary inflorescences for several years before a terminal inflorescence, others produce a terminal inflorescence in their first year (Lesica & Shelly, 1995; Lesica & Young, 2005). A similar system is seen in common foxglove, *Digitalis purpurea* (Scrophulariaceae), which is predominantly biennial or perennial‐iteroparous, but can be facultatively semelparous if resource availability in the first year is high (Sletvold, 2002). Facultative semelparity has also been observed in capelin (Christiansen et al., 2008; Loïc et al., 2012), squid, soil microarthropods (Siepel, 1994), dasyurid marsupials (Kraaijeveld, Kraaijeveld‐Smit, and Adcock, 2003; Martins, Bonato, Silva & Reis, 2006), and in the flowering plants *Ipomopsis aggregata* (Silvertown & Gordon, 1989) and *Cynoglossum officinale* (Williams, 2009). Some facultatively semelparous species show a continuous range of types of reproductive episode, rather than discretely fatal or nonfatal ones. *Erysimum capitatum* (Brassicaceae) produces multiple reproductive episodes in environments where water is plentiful; however, where water is scarce, it expresses a semelparous strategy (Kim & Donohue, 2011).

#### 3.2.3 Multiple modes of parity

The realization of multiple modes of parity at once is a major source of confusion for mathematical models that predict a single optimal value for all individuals, regardless of whether they are all supposed to express an annual‐semelparous or perennial‐iteroparous habit. The range of different modes of parity expressed need not be dramatic and may be due to phenotypic plasticity, but, as a consistent response to environmental triggers, even small differences in the degree of concentration of reproductive effort should significantly affect fitness. In many cases, the simultaneous realization of multiple modes of parity occurs because different individuals in a population express a continuous range of modes of parity— for example, some annual plants reproduce over a long timescale, others complete reproduction over only a few days (e.g., Hughes & Simons, 2014c). Such continuous differences in mode of parity can occur both: (1) among individuals; or (2) within the reproductive episode of a single individual.

Strong empirical evidence of multiple modes of parity realized at once is found in sea beets (Beta spp., Amaranthaceae), which display reproductive strategies along “a gradient from pronounced iteroparity to pronounced semelparity” (Hautekèete et al., 2001, p. 796). Interestingly, the production of multiple modes of parity is elicited as an adaptive response to variable selective pressures faced by these species (e.g., predation and disturbance). High levels of environmental stress cause individuals to trade off future fecundity for increased immediate reproductive effort, resulting in a parity gradient tending to semelparity wherever environmental stress becomes intense (Hautekèete, Piquot, and Van Dijk, 2001, 2009). This pattern is consistent with the prediction that higher current reproductive effort can prevent organisms from being exposed to uncertain or risky environments (Rubenstein, 2011; Trumbo, 2013; Vahl, 1981; Williams, 1966). Similar trade‐offs have been observed in *Yucca whipplei* (Huxman & Loik, 1997), *Chusquea ramosissima* (Montti, Campanello & Goldstein, 2011), and *Onopordum illyricum* (Rees, Sheppard, Briese & Mangel, 1999). Populations of *Lobelia inflata* are also capable of producing a range of different modes of parity, from a nearly instantaneous annual‐semelparity, where plants produce many similar flowers quickly and simultaneously, to (nonadaptive) facultative biennial‐iteroparity, where as much as half of all reproductive effort is invested in a second reproductive episode. The time of initiation of reproduction strongly predicted which of these strategies is realized (Hughes & Simons, 2014b,c).

Many insect species are also capable of displaying a range of modes of parity among individuals (Trumbo, 2013). In the assassin bug (*Atopozelus pallens*), females deposit eggs in small clutches, approximately every two days. However, the number of clutches—and hence how prolonged this reproductive episode is—varies substantially (Tallamy, Walsh & Peck, 2004). Similarly, female European earwigs (*Forficula auricularia*) show continuous variation in clutch size and can even become semelparous by laying only a single one (Meunier et al., 2012; Ratz, Kramer, Veuille & Meunier, 2016). Most insects showing variation in the number of clutches produced do so in response to abiotic cues, particularly temperature and day length (Bradshaw, 1986). This behavior can also be found in ascidians (Grosberg, 1988) and semelparous mammals (Mills et al., 2012; Wolfe, Mills, Garkaklis & Bencini, 2004).

Phenotypic plasticity within a reproductive episode of a single individual is noticeable when a semelparous organism displays a changing reproductive strategy—varying along the continuum of parity—that cannot be attributed to developmental, environmental, or architectural constraints (Diggle, 1995, 1997). This pattern is more difficult to detect than phenotypically plastic strategies that differ between individuals, but in many systems observable differences exist between the “packaging” of reproductive effort, resulting in adaptive variation in phenology or offspring quality through time. This can also be difficult, because—since they reproduce only once—semelparous organisms are expected to show high reproductive effort (Bonser & Aarssen, 2006). However, the development of fruits of the semelparous plant *Lobelia inflata* varied continuously; in this system, late fruits showed accelerated phenology and higher offspring quality relative to early fruits. This pattern, which indicated that more reproductive effort was invested in later fruit, shows that *L. inflata* does not “reproduce once” but dynamically allocates reproductive effort throughout a sequence of repeated fruiting events (Hughes & Simons, 2014a, 2015). Likewise, in populations of the semelparous plant *Centaurea corymbosa*, plants showed highly variable life cycles—dynamically varying the proportion of reproductive effort allocated to sequential flowers—depending on environmental conditions and crowding (Acker, Robert, Bourget & Colas, 2014).

### 3.3 Evolutionary transitions between modes of parity are ubiquitous

Transitions between different strategies along the semelparity–iteroparity continuum are common throughout the tree of life. Furthermore, modes of parity appear to be evolutionarily labile within species, and many species show significant intraspecific differences in the expression of parity (Hughes & Simons, 2014a,c; Maltby & Calow, 1986); these changes are often due to differences in the genetic regulation of the traits underlying modes of parity. While some clades consistently display a narrower range of modes of parity (e.g., placental mammals are typically iteroparous and have at most tens of reproductive bouts in their lives, many others show considerable variability (see Table 1 for data from plant orders). Among cephalopoda (Mollusca), *Loligo opalescens*,* Octopus vulgaris*,* O. mimus*, and *O. cyanea* display extreme semelparity (Ikeda, Sakurai & Shimizaki, 1993; Rocha et al., 2001), while *Nautilus* spp. show extreme perennial‐iteroparity (Ward, 1983, 1987). However, other cephalopods, including *Octopus chierchiae*,* Sthenoteuthis oualaniensis*, and *Dosidicus gigas* show varying degrees of facultative iteroparity (Laptikhovsky, 1998, 1999; Nesis, 1996; Rocha et al., 2001), while still others, including *Sepia officinalis*,* Loligo vulgaris*,* L. bleekeri*,* L. forbesi*, and *Ilex coindetii* show facultative semelparity, and, in the case of *S. officinalis*, a strikingly variable duration of reproduction (Baeg, Sakurai & Shimazaki, 1993; Boletzky, 1987, 1988; Gonzalez, 1994; Gonzalez & Guerra, 1996; Melo & Sauer, 1999; Rocha & Guerra, 1996). Furthermore, in many of these species key traits—such as the timing and duration of reproduction—show substantial dependence on environmental effects (Rocha et al., 2001). Similar lability in these traits is also present in other clades, including both angiosperms and animals (Crespi & Teo, 2002; Hautekèete, Piquot, and Van Dijk, 2001; Maltby & Calow, 1986; Tallamy & Brown, 1999; Varela‐Lasheras & Van Dooren, 2014). Therefore, since transitions from mode of parity to another have occurred throughout the tree of life, a continuous understanding of parity may clarify the relationship between life‐history strategy and speciation.

## 4 UNDERSTANDING THE EVOLUTION OF PARITY AS A CONTINUOUS TRAIT

What changes should be made in light of the evidence that parity is a continuous trait? In this section, I will focus on three main recommendations. First, I provide a short discussion of how seasonality should not be conflated with mode of parity. Second, I discuss the necessity of developing new mathematical modeling approaches that treat parity as a continuous variable. This is not simple, since parity itself is a composite trait, and relies on the coordination of many biological functions at once. Third, I discuss why ecologists should ground future studies of adaptive life‐history strategies in mechanistic details derived from genetic studies of continuously varying life‐history traits underlying reproduction and, consequently, parity. These recommendations should improve both the validity and reliability of predictive models of life‐history evolution, while simultaneously providing a framework for interpreting empirical findings regarding the expression of reproductive effort through time.

### 4.1 Seasonality and mode of parity

One major implication of treating parity as a continuous variable is that this reconception allows us to distinguish between parity and seasonality. Parity describes the concentration or diffusion of reproductive effort in time, which is distinct from the question of seasonal reproduction—that is, how organisms should distribute reproductive effort among seasons, when seasonal cycles determine the favorability of establishment, growth, and reproductive conditions (Bulmer, 1994; Calow, 1979; Charnov & Schaffer, 1973; Cole, 1954; Evans et al., 2005; Ranta, Tesar, Alaja & Kaitala, 2007; Schaffer and Gadgil (1975); Schaffer, 1974b; Young, 1981). It is, of course, clear that seasonality is related to parity. Insofar as an annual‐semelparous organism is defined by the fact that it has a single reproductive episode that occurs within one year, it is likely to experience selection for strategies that optimize its reproductive schedule relative to season‐specific environmental effects; this means that an annual‐semelparous organism is more likely to show predictable seasonal patterns than a perennial‐iteroparous congener that can escape a poor season by overwintering. However, the explanatory power of such seasonal adaptations may be much weaker when we compare a fast‐reproducing semelparous organism with a slower‐reproducing semelparous congener, or when we compare an iteroparous strategy where reproductive effort is distributed over two seasons with another where reproductive effort is distributed among ten seasons. Seasonal effects are likely to supervene on reproduction whenever regular intervals occur that have an impact on the favorability of reproduction. Thus, it may be more fruitful to understand annuality and perenniality as strategies defined by the “digitization” of reproduction in response to seasonality. The advantage of this approach is that it makes it easier to understand flexible life histories, regardless of whether a species is semelparous or iteroparous.

There is widespread empirical evidence that seasonality and parity can vary independently. One common pattern is integer changes in voltinism among organisms that share a common mode of parity. For example, the Muga silkworm (*Antheraea assamensis*) is semelparous and multivoltine throughout its natural range (from India to Borneo). This species produces up to six generations per year, with the number of reproductive cycles depending on length of the season (Ghorai, Chaudhuri & Senapati, 2009; Singh & Singh, 1998). However, the closely related Chinese tussar silkmoth, *Antheraea pernyi*, is bivoltine at the southern margins of its range, but is univoltine in northern China and Korea. Moreover, this continuous variation in voltinism along an ecological cline is due to continuous variation in environment‐dependent biogenic monoamine production in the brains of diapause pupae (Fukuda, 1953; Liu, Li, Li & Qin, 2010; Matsumoto & Takeda, 2002). Life histories also vary continuously among populations of the wild silkmoth (*Bombyx mandarina*) and its domesticated counterpart (*Bombyx mori*), where populations in colder climates (e.g., European Russia) are univoltine, whereas those in China and Korea are bivoltine or multivoltine (Xia et al., 2009). Similar examples can also be found in crucifers (Springthorpe & Penfield, 2015; Williams & Hill, 1986), orchids (Chase, Hanson, Albert, Whitten & Williams, 2005), freshwater mollusks (Mackie & Flippance, 1983; McMahon & Bogan, 2001), and Centaurea (Asteraceae; Acker et al., 2014), among others. In each of these systems, a distinct continuum of reproductive strategies despite the supervening effect of seasonality is readily observable. Additionally, new models are being developed that consider generation length independently from parity (Waples, 2016). Thus, we can easily tease apart the question of whether reproduction is concentrated in time—that is, whether a given species is semelparous—from the question of whether seasonality requires that, in temperate climates, late‐reproducing individuals should enter diapause rather than reproduce immediately.

### 4.2 Mathematical models of parity

A second problem facing life‐history theory is the challenge of extending the logic of existing life‐history models to account for the continuity of modes of parity. Although empirical studies of many taxa support the continuous conception of parity, the evolution of different modes of parity from one another has generally been explained by demographic models that compare the special case where annual‐semelparous and perennial‐iteroparous strategies have different demographic implications. This makes it relatively difficult to even generate predictions for organisms whose reproductive behavior does not show an explicitly annual‐semelparous or perennial‐iteroparous life history, or for species or populations that display continuous differences in parity. Generalizing these models to provide quantitative predictions for such situations should thus be an important goal for life‐history theory. A newer model should, as an axiom, treat parity as a continuous trait and should be able to explain both the evolution of semelparous strategies from iteroparous ones (or vice versa) as well as the adaptive value of intermediate modes of parity.

These new models will have to build on and learn from a considerable body of existing models detailing the eco‐evolutionary dynamics of semelparous and iteroparous life‐history strategies. Early conceptual and mathematical models of optimal semelparous reproduction were generally simple and deterministic and were designed to predict a single “threshold” value that optimized life‐history characters such as size at first reproduction (Bell, 1980; Young, 1981). Threshold models of this type include senescence‐threshold models based on the Penna aging model (Piñol & Banzon, 2011), as well as development‐threshold models such as age‐structured life‐history models. Age‐structured models treat age at reproduction, and hence parity, as a discrete variable, and assess the evolutionary consequences of the degree of overlap between juvenile (i.e., prereproductive) and adult (reproductive) classes in a population (Wikan, 2012). Among the best known of these are Leslie models, which predict either few evolutionary stable states for semelparous organisms (Cushing, 2009, 2015; Cushing & Henson, 2012; Cushing & Stump, 2013) or even that populations should consist entirely of individuals of a given age class (Rudnicki & Wieczorek, 2014). Still other threshold models make similar predictions for survival traits (Da‐Silva, Martins, Bonato & Dos Reis, 2008).

Some of the assumptions made by threshold models may be resolved by incorporating a wider range of possible life histories. For instance, Rees et al. (1999) showed that those deterministic age‐structured models, which rest on the assumption that parity is discrete, consistently overestimate time at first reproduction in monocarpic plants. This result is the problem of the discrete conception writ large: Empirical data does not conform to model predictions because empirically, the concentration of reproductive effort in time is not as extreme as would be predicted by if the annual‐semelparous life history were as extreme as it was predicted to be (see also Marshall and Keough, 2007). Such variability—caused by developmental plasticity and stochastic variation in the timing of cues—confounds threshold models, in which semelparous reproduction is held to be optimized closely within a given environment and has therefore prompted the formulation of new modeling approaches that consider a range of semelparous strategies in response to environmental heterogeneity (reviewed in Metcalf et al., 2003).

Recent mathematical models also fall into several types, each with a particular ecological focus. Integral projection models, which incorporate random fluctuations in environmental parameters related to reproduction, were developed to more accurately predict time to first reproduction and size at reproduction, both in iteroparous species (e.g., Kuss, Rees, Aegisdottir, Ellner & Stocklin, 2008) and in semelparous species with a prolonged semelparous reproductive episode (Ellner & Rees, 2006; Rees, Sheppard, Briese, and Mangel, 1999; Sletvold 2005). Time‐lagged integral projection models attempt to account for the temporal discounting of reproductive value as well as size‐specific effects on reproductive effort (Kuss et al., 2008). Newer age‐structured stochastic models incorporate continuous variation in life‐history traits to predict optimal timing of reproduction; while these resemble earlier models that treat parity wholly as a discrete variable, the life‐history traits in these models are treated continuously (Davison & Satterthwaite, 2016; Oizumi, 2014; Oizumi & Takada, 2013).

Several recent models have been developed to predict reproductive trait values given other (measured or measurable) life‐history parameters. This modeling methodology is intuitive and compatible with the idea that parity is a continuous trait. For example, Kindsvater et al. (2016) used a stage‐structured model to assess the degree to which trait covariation constrained life‐history adaptation in salmonids. Other kinds of data‐driven models fall into two main types: (1) models that highlight the importance of phenotypically plastic reaction norms as maximizing fitness despite stochastic variability in environment (e.g., Burd et al., 2006); and (2) models that emphasize the innate variability in reproductive characters within species (Austen, Forrest & Weis, 2015; Drouineau, Rigaud, Daverat & Lambert, 2014). Both of these ideas may be useful in modeling selective pressures on a continuum of modes of parity. Moreover, rather than using a single model to characterize semelparous investment in flowers and offspring, authors are now proposing a “meta‐modeling” approach to annual plant reproduction, recognizing that semelparous reproduction can be fine‐tuned by natural selection through phenotypic plasticity (Hughes & Simons, 2014a).

Because parity may be most fruitfully understood as the concentration of reproductive effort in time, another class of model that may prove to be useful is the dynamic state variable model (DSVM). DSVMs are powerful dynamic optimization models used to characterize mechanistic relationships in ecology and have the benefit of being able to be solved computationally (Clark & Mangel, 2000). Developing a DSVM can offer insight into the relative impact of underlying causal processes (in this case, the underlying patterns of genetic regulation of reproductive traits) on the concentration of reproductive effort, and, ultimately, on a state variable of interest (in this case, total plant fitness). Because the model follows the value of a state variable, the effects of multiple fitness components can be considered at once. Moreover, by parameterizing a DVSM with phenotypic data, ecologists can determine the additive and multiplicative contributions of variation at different gene loci, or between related phenotypes of interest. This is an important advantage insofar as continuous models of parity should, where possible, include mechanistic detail. DSVMs are compatible with this approach: They can integrate a wide range of functional, spatial, structural, behavioral, or environmental limitations constraining investment in reproduction and can generate testable predictions by determining optimal reproductive decision schedules (Peterson & Roitberg, 2010; Skubic, Taborsky, McNamara & Houston, 2004; Yerkes & Koops, 1999). Although the specification of mathematical models is complex, and a thorough articulation and validation of a mathematical model of continuously varying parity is beyond the scope of this review, this approach offers hope for a simple, iterative improvement of the discrete‐conception threshold and age‐class models that have been validated in the past.

### 4.3 Molecular regulation of parity

The third implication of understanding parity as a continuously varying trait is that life‐history models should be rooted in mechanistic detail, and identifying the mechanistic basis of different modes of parity (e.g., the contributions of individual genes and/or molecular pathways responsible for initiating and continuing reproduction) should be an important priority for evolutionary ecology. These models should supplement and extend the theoretical predictions of extant conceptual models. Integrating theoretical ecology with molecular biological data was not possible when early life‐history models were developed, but since parity is determined by the onset and completion of reproductive episodes, and recent advances in molecular ecology have made it possible to understand the physiological and genetic basis of the timing of these events in many systems, developing an approximate integration of molecular detail and theoretical explanation is, in many systems, an achievable goal. Numerous examples of continuously expressed physiological processes result in continuous patterns of reproduction, and hence support the continuous conception of parity (Table 3). In this section, I will briefly explain how parsing out the contributions of a single gene can improve our understanding of how modes of parity can vary continuously. To do so, I will discuss an important example: the control of the initiation of flowering in response to vernalization as it is regulated by *FLOWERING LOCUS C* (*FLC*) and its orthologues in the Brassicaceae.

**Table 3:** Genes and QTLs regulating continuously expressed traits linked to parity

Study Organism	Clade	Gene/QTL	Traits	References
*Actinidia chinensis*	Angiosperm	carotenoid cleavage dioxygenase 8 (CCD8)	Branch development; timing of senescence	Ledger et al. (2010)
*Aedes aegypti, A. albopictus*	Insect	cytochrome P450 gene 6Z6 (CYP6Z6), 6N12 (CYP6N12), and M9 (CYP9M9)	Juvenile mortality	Kim and Muturi (2012)
*Arabidopsis lyrata*	Angiosperm	LG3, LG4	Developmental timing of reproductive transitions; branching	Leinonen et al. (2013), Remington et al. (2013, 2015)
*Arabidopsis thaliana*	Angiosperm	Early day‐length insensitive (EDI)	Response to vernalization; timing of flowering	Alonso‐Blanco et al. (1998)
*Arabidopsis thaliana*	Angiosperm	FLC	Timing of initiation of reproduction	Bastow et al. (2004), Caicedo et al. (2004), Kim et al. (2007), Aikawa et al. (2010), Deng et al. (2011)
*Arabidopsis thaliana*	Angiosperm	FRI	Timing of initiation of reproduction	Le Corre et al. (2002), Stinchcombe et al. (2004), Shindo et al. (2005), Méndez‐Vigo et al. (2011)
*Arabidopsis thaliana*	Angiosperm	DOG1	Seed dormancy; timing of initiation of reproduction	Chiang et al. (2011, 2013)
*Arabidopsis thaliana; Pisum sativum*	Angiosperm	More axillary growth (MAX4); Ramosus1 (RMS1)	Axillary shoot outgrowth; timing of outgrowth	Sorefan et al. (2003)
*Arabis alpina*	Angiosperm	PEP1	Timing of initiation of reproduction; perenniality	Wang, Farrona, Vincent, Fornara, et al. (2009), Albani et al. (2012), Castaings et al. (2014)
*Brachionus plicatilis*	Rotifer	small heat shock protein 1 (shsp‐1), shsp‐2, shsp‐3, shsp‐4	Dormancy; juvenile mortality	Denekamp et al. (2011)
*Colias eutytheme, C. philodice*	Insect	phosphoglucose isomerase (PGI)	Lifespan, fecundity	Watt (1983), Watt et al. (1983)
*Conregonus clupeaformis*	Fish	*Salmo salar* zonadhesin‐like	Fecundity	Nolte et al. (2009)
*Drosophila melanogaster*	Insect	Met	Fecundity, timing of initiation of reproduction	Flatt and Kawecki (2004)
*Drosophila melanogaster*	Insect	Juvenile hormone (JH), TOR	Lifespan, timing of transition to adulthood	Tatar, Chien, and Priest (2001), Tatar, Bartke, and Antebi (2003), Kapahi et al. (2004), Katewa and Kapahi (2011)
*Gadus morhua*	Fish	Growth hormone 1	Timing of juvenile maturation	Hemmer‐Hansen et al. (2014)
*Gryllus firmus*	Insect	Juvenile hormone (JH)	Fecundity	Zera and Huang (1999), Zhao and Zera (2002)
*Haliotis refescens*	Mollusk	engrailed, aragonite protein 24k Da (ap24)	Juvenile mortality	Zippay et al. (2010)
*Heliocidaris erythrogamma*	Echinoderm	Abopec, Brn1, Brn2, Brn4	Feeding behavior; timing of initiation of reproduction	Israel et al. (2016)
*Hordeum vulgare*	Angiosperm	QSD1	Seed dormancy	Sato et al. (2016)
*Ichthyomyzon castaneus, I. fossor*	Lamprey	insulin‐like growth factor 1 receptor (igf1r), cytochrome c oxidase subunit III (coIII)	Fecundity	Spice et al. (2014)
*Lactuca sativa*	Angiosperm	DOG1	Seed dormancy; timing of initiation of reproduction	Huo et al. (2016)
*Miletaea cinxia*	Insect	phosphoglucose isomerase (PGI)	Lifespan, fecundity	Klemme and Hanski (2009), Saastamoinen et al. (2009)
*Miletaea cinxia*	Insect	troponin‐t (TNT)	Timing of transition to adulthood	Marden et al. (2008)
*Mimulus guttatus*	Angiosperm	More axillary growth (MAX)	Axillary shoot outgrowth; timing of outgrowth	Baker et al. (2012)
*Mimulus guttatus; M. nasutus*	Angiosperm	–	Timing of reproduction; reproductive allocation	Hall et al. (2006)
*Oncorhynchus kisutch, O. keta, O. gorbuscha*	Fish	Clock, Cytochrome	Timing of juvenile maturation	O'Malley et al. (2010)
*Oncorhynchus mykiss*	Fish	Omy5 loci, including OC6, OC8, OC14, OC20, OC21, and OC30	Anadromy, timing of smoltification	Nichols et al. (2008), Pearse et al. (2014)
*Oncorhynchus mykiss*	Fish	Clock	Spawning time	Leder et al. (2006)
*Oncorhynchus mykiss*	Fish	One3ASC, One19ASC	Biannual spawning	Colihueque et al. (2010)
*Oncorhynchus mykiss, Oncorhynchus kitsuch*	Fish	17a, 20b‐dihydroxy‐4‐prengnen‐3‐one	Timing of senescence	Barry et al. (2010)
*Petromyzon marinas, Lampetra appendix*	Lamprey	Gonadotropin‐releasing hormone‐I (GnRH‐I) and Gonadotropin‐releasing hormone‐III (GnRH‐III)	Timing of transition to adulthood	Youson et al. (2006)
*Petunia hybrida*	Angiosperm	Decreased apical dominance 1 (DAD1), MAX1, MAX2, CCD7, CCD8	Axillary shoot outgrowth; timing of and reproductive effort allocated to floral development	Snowden (2005), Drummond (2012)
*Rhagoletis pomonella*	Insect	jetlag (jet), clockwork orange (cwo), PAR‐domain protein 1 (Pdp1)	Timing of diapause termination	Ragland et al. (2011)
*Sarcophaga crassipalpis*	Insect	heat shock protein 23 (Hsp23), Hsp70, Hsp90, lipid storage protein (LSP)‐1, LSP‐2	Timing of diapause termination; timing of reproduction; reproductive effort	Rinehart et al. (2000, 2007), Hahn et al. (2008)
*Thlaspi caerulescens*	Angiosperm	Thlc1, Thlc2, Thlc3	Fecundity, timing of initiation of reproduction	Jiménez‐Ambriz et al. (2007)

In *Arabidopsis*, continuous variation in parity—that is, the timing of floral initiation and the duration of flowering—is determined by continuous expression of flowering‐time genes, including *FLOWERING LOCUS T* (*FT*) (Imaizumi & Kay, 2006; Kardailsky, 1999; Kotake, Takada, Nakahigashi, Ohto & Goto, 2003; Simon, Rühl, Montaigu, Wötzel & Coupland, 2015; Turck, Fornara & Coupland, 2008; Yanovsky & Kay, 2002), *FRIGIDA* (*FRI*) (Johanson et al., 2000; Le Corre, Roux & Reboud, 2002; Michaels, Bezerra & Amasino, 2004; Schläppi, 2006; Shindo et al., 2005; Stinchcombe et al., 2004), *FLOWERING LOCUS C* (Amasino, 1996; Bastow et al., 2004; Chiang, Barua, Kramer, Amasino & Donohue, 2009; Coupland, 1995; Imaizumi & Kay, 2006; Kim et al., 2007; Michaels & Amasino, 1999; Michaels, He, Scortecci, and Amasino, 2003; Michaels et al., 2004; Sheldon, Rouse, Finnegan, Peacock & Dennis, 2000), *GIGANTEA* (*GI*) (Fowler et al., 1999; Jung et al., 2007; Mizoguchi et al., 2005), and *CONSTANS* (*CO*) (Koornneef, Alonso‐Blanco, Peeters & Soppe, 1998; Putterill, Robson, Lee, Simon & Coupland, 1995; Redei, 1962; Samach et al., 2000; Suárez‐López et al., 2001; Valverde et al., 2004). Through different pathways, *GI* and *CO* activate the floral integrator gene *FLOWERING LOCUS T* (*FT*), which transcribes a protein that activates floral identity genes in the shoot apical meristem (Tiwari et al., 2010; Turck et al., 2008). In contrast, *FLC*—along with *FRI*, which regulates *FLC* transcription—represses flowering until exposure to cold silences its expression.

Although much is known about flowering in the Brassicaceae, here I concentrate on *FLC*, since continuous differences in *FLC* expression cause continuous variation in the duration and timing of semelparous‐annual reproduction in *A. thaliana* (Burghardt, Metcalf, Wilzcek, Schmitt & Donohue, 2015; Wilzcek et al., 2009). This variation causes continuous differences in parity. For instance, throughout Europe, parity in wild populations of *Arabidopsis thaliana* is strongly determined by climate and/or latitude: Toward the colder margins of its range, in northern Finland, plants show a fast‐cycling summer semelparous‐annual life history, while populations near the Mediterranean show a winter‐annual life history, and populations in intermediate locations (e.g., the UK) display intermediate life histories (Ågren & Schemske, 2012; Méndez‐Vigo, Picó, Ramiro, Martínez‐Zapater & Alonso‐Blanco, 2011; Thompson, 1994). Laboratory studies have identified *FLC* as a gene responsible for this life‐history variation. For example, Wilzcek, Roe, Knapp, Cooper, Martin, Muir, Sim et al. (2009) introgressed a functional *FRI* allele into *A. thaliana* ecotypes with nonfunctional alleles. They predicted that this genetic modification—which causes the upregulation of *FLC*—would see plants transition from a summer‐annual to winter‐annual life history. Instead, plants with functional *FRI* alleles flowered only 10 days later than those with nonfunctional *FRI* alleles, causing the authors to note that their results “suggest that *A. thaliana* ecotypes cannot simply be divided into two discrete classes of winter‐annual and rapid‐cycling genotypes. Rather, most ecotypes may be capable of both life histories” (p. 933). This prediction is consistent with recent data from studies of the impact of *FLC* on the life histories of *A. thaliana* ecotypes sourced from different parts of its native range. While populations varying at the *FLC* locus show substantial local adaptation with respect to important life‐history traits—including those, such as length of duration of reproduction, which underlie mode of parity—most ecotypes adopt new life histories when translocated to radically different environments (Ågren, Oakley, Lundemo & Schemske, 2016; Dittmar, Oakley, Ågren & Schemske, 2014). In this way, studying the regulation of *FLC* also provides a useful study of how seasonality can supervene on reproduction, since plants from the same genetic background can show starkly different life histories when subjected to different seasonal schedules (Ågren & Schemske, 2012; Postma & Ågren, 2016).

Where the prevalence of different *FLC* alleles differs between populations grown in similar environments, differences in *FLC* expression can result in different flowering phenologies, and even different modes of parity (Banta & Purugganan, 2011; Johanson et al., 2000; Michaels, Bezerra, and Amasino, 2004; Schläppi, 2006). This probably an adaptive response; life‐history models of the natural genetic variation at the *FLC* and *FRI* loci have shown that *FLC* expression explains a relatively high level of variation in fitness (Burghardt et al., 2015; Donohue, Burghardt, Runcie, Bradford & Schmitt, 2014; Springthorpe & Penfield, 2015). Moreover, empirical studies suggest that such fitness differences may account for the latitudinal cline in *Arabidopsis* life history found in natural populations (Caicedo, Stinchcombe, Olsen, Schmitt & Purugganan, 2004). Thus, it seems that local adaptation of different modes of parity results from populations experiencing stabilizing selection for climate‐appropriate *FLC* alleles (Postma & Ågren, 2016).

While fitness differences are tightly linked to phenotypic variation, major plant phenotypes such as flowering phenology are highly plastic in *Arabidopsis*. The genetic and epigenetic regulation of *FLC* regulates the timing of important life‐history transitions, determining a plant's reproductive schedule, and hence its mode of parity (Albani & Coupland, 2010; Turck & Coupland, 2014). However, environmental factors such as seed maturation and germination temperatures can interact with plant genotypes to produce “clusters” of phenotypically similar, yet genotypically distinct, plant phenologies (Burghardt, Edwards & Donohue, 2016). Environmental variation can therefore facilitate the adoption of multiple flowering phenologies (e.g., summer annual, winter annual, rapid cycling)—and thus modes of parity—from a single *Arabidopsis* ecotype (Méndez‐Vigo et al., 2011; Simpson & Dean, 2002).

Understanding how the *FLC* locus regulates the concentration of reproductive effort in time is a conceptually important example of how genetics can be an important source of new data for next‐generation models of the evolution of modes of parity. *FLC* is not an isolated example of a single locus strongly influencing parity. Although a comprehensive description of all genes linked to parity in all species is beyond the scope of this article, a few notable examples from a variety of well‐studied taxa are presented in Table 3. Genes linked to traits underlying parity, including reproductive maturation, stress response, reproductive phenology, and senescence, have also been the subject of ongoing research (Albani, Castaings, Wötzel, Mateos & Wunder, 2012; Amasino, 2009; Bastow et al., 2004; Blümel, Dally & Jung, 2015; Castaings, Bergonzi, Albani, Kemi & Savolainen, 2014; Costantini, Battilana, Lamaj, Fanizza & Grando, 2008; Danon, Delorme, Mailhac & Gallois, 2000; Eulgem, Rushton, Robatzek & Somssich, 2000; Finch & Rose, 1995; Garcia De Leaniz, Fleming, Einum, Verspoor & Jordan, 2007; Hall, Luquez, Garcia, St Onge & Jansson, 2007; Kenyon, 2011; McBlain, Hesketh & Bernard, 1987; McCormick, Tsai & Kennedy, 2011; Partridge, 2010; Rion & Kawecki, 2007; Schneider & Wolf, 2008; Selman & Withers, 2011; Sheldon et al., 2000; Thomas, 2013; Thomas, Huang, Young & Ougham, 2009; Tower, 1996; Wang, Cheng, Leng, Wu & Shao, 2015; Wang, Farrona, Vincent, Fornara, et al. 2009; Wang, Farrona, Vincent, Joecker, et al. 2009; Xin, Qiu, Shan, Shan & Liu, 2008).

## 5 CONCLUSIONS

We still know far too little about why the evolutionary transition from semelparity to iteroparity (or vice versa) is as common as it is, or under which ecological conditions intermediate strategies—such as facultative semelparity—will thrive. Models rooted in the conception of parity as a binary trait do a good job of accounting for the fitness differences between discrete semelparous‐annual and iteroparous perennial alternative strategies, and, even when they do not make accurate quantitative predictions, they have heuristic value (e.g., they permit the consideration of the impact that factors such as density dependence and environmental stochasticity will have on parity). However, systems characterized by only these possibilities—and no others—are special cases, and thus their insights. In most cases, the life‐history question at hand is subtle: Why does a given species evolve a facultative strategy, or why does another show intraspecific variation in the length of its semelparous reproductive episode?

1. The main conclusion of this work is that parity should be understood as the concentration of reproductive effort in time and should therefore be treated as a continuous trait rather than a discrete one. This generalization of parity offers several notable advantages for life‐history theory. First, treating parity as a continuous trait allows us to treat parity as a distinct life‐history syndrome, itself the result of correlated selection on a suite of continuously varying traits affecting the concentration of reproductive effort in time, and which may show finely graded correlated variation within species or populations. This is advantageous because parity is a composite trait, and the act of reproducing at a given time, for a given duration, etc. involves the recruitment and coordination of many independent traits, each of which may affect the expression of others. A similar integrative approach has proven to be valuable in studying other multifactorial composite traits, such as dispersal and risk spreading (Buoro & Carlson, 2014). Second, whether they share a common genetic basis or not, obvious or visible life‐history characters may not be primary targets of selection, and evolution of such traits may occur as an epiphenomenon of selection on (one or many) other apparent or nonapparent underlying traits.
2. Next, the question of parity should be separated from the question of seasonality; this is a source of abundant confusion. The question of the concentration of reproductive effort within a reproductive episode is simply not the same as the question of the optimal pattern of the distribution of risk in and among seasons. These questions are undoubtedly related: Reproductive characters of long‐lived semelparous species are generally easier to model than characters of short‐lived species, and while environmental heterogeneity plays an important role determining the optimal allocation of reproductive effort in annual‐semelparous species, long‐lived semelparous species can afford to be “choosier” about when they reproduce, and therefore have been shown to more closely approximate model predictions. This may be especially true when, as in many long‐lived perennial‐iteroparous species, the relationship between age and cost of reproduction is nonlinear. Thus, developing models that accurately model the fitness dynamics of short‐lived semelparous species should be a priority.
3. Third, life‐history theorists should work to extend the insights from abstract discrete‐conception conceptual and mathematical models to next‐generation models that treat parity as a continuously varying trait. Considering only annual‐semelparity and perennial‐iteroparity as discrete alternatives, although a useful simplification for many models, is biologically accurate only in a limited number of special cases, and the continuous conception of parity is more likely to approximate the eco‐evolutionary dynamics of natural systems that show intraspecific or plastic variation in the expression of parity.
4. Lastly, treating parity as a continuous variable that represents a syndrome of associated traits makes it easier to integrate life‐history studies with mechanistic details deriving from molecular ecology, insofar as composite life‐history traits such as parity are unlikely to be the result of a simple presence or absence of a single gene or allele. Instead, parity is likely to be the product of complex systems of genetic, translational, and post‐translational regulation. Systems in which discrete modes of parity are found may therefore reflect those cases where continuous variation in underlying traits is masked by the supervening effect of developmental thresholds that can trigger reproduction (or not).

## CONFLICT OF INTEREST

None declared.

## AUTHOR CONTRIBUTIONS

PWH was responsible for researching and writing this manuscript.
